# If you feed them, they will come: A prospective study of the effects of complimentary food on attendance and physician attitudes at medical grand rounds at an academic medical center

**DOI:** 10.1186/1472-6920-7-22

**Published:** 2007-07-12

**Authors:** Colin M Segovis, Paul S Mueller, Melissa L Rethlefsen, Nicholas F LaRusso, Scott C Litin, Ayalew Tefferi, Thomas M Habermann

**Affiliations:** 1 Division of General Internal Medicine, Mayo Clinic, 200 First Street SW, Rochester, MN 55905, USA; 2 Learning Resource Center, Mayo Clinic, 200 First Street SW, Rochester, MN 55905, USA; 3 Division of Gastroenterology and Hepatology, Mayo Clinic, 200 First Street SW, Rochester, MN 55905, USA; 4 Division of Hematology, Mayo Clinic, 200 First Street SW, Rochester, MN 55905, USA

## Abstract

**Background:**

Evidence suggests that attendance at medical grand rounds at academic medical centers is waning. The present study examined whether attendance at medical grand rounds increased after providing complimentary food to attendees and also assessed attendee attitudes about complimentary food.

**Methods:**

In this prospective, before-and-after study, attendance at medical grand rounds was monitored from September 25, 2002, to June 2, 2004, using head counts. With unrestricted industry (eg, pharmaceutical) financial support, complimentary food was provided to medical grand rounds attendees beginning June 4, 2003. Attendance was compared during the pre-complimentary food and complimentary food periods. Attitudes about the complimentary food were assessed with use of a survey administered to attendees at the conclusion of the study period.

**Results:**

The mean (± SD) overall attendance by head counts increased 38.4% from 184.1 ± 90.4 during the pre-complimentary food period to 254.8 ± 60.5 during the complimentary food period (*P *< .001). At the end of the study period, 70.1% of the attendee survey respondents indicated that they were more likely to attend grand rounds because of complimentary food, 53.6% indicated that their attendance increased as a result of complimentary food, and 53.1% indicated that their attendance would decrease if complimentary food was no longer provided. Notably, 80.3% indicated that food was not a distraction, and 81.7% disagreed that industry representatives had influence over medical grand rounds because of their financial support for the food.

**Conclusion:**

Providing free food may be an effective strategy for increasing attendance at medical grand rounds.

## Background

Medical grand rounds (MGR) is a central teaching activity in US departments of medicine at academic medical centers. However, attendance at MGR by faculty, fellows, and residents appears to be waning even though the perceived quality of MGR is increasing [1-3]. This decrease in attendance, coupled with the considerable resources (eg, financial resources and time) invested in this activity, should cause MGR planners and continuing medical education (CME) providers to question why attendance is waning.

Moore [4] described a 6-level approach for evaluating the value of CME. Each level is associated with an outcome: participation/attendance, satisfaction, learning, performance, patient health, and population health. The present study focuses on measuring attendance as an outcomes measure. On the basis of Moore's model, increasing attendance is a legitimate CME goal.

One commonly used strategy to increase attendance at MGR is to provide complimentary food [3]. Observational and anecdotal data indicate that complimentary food can increase attendance at meetings and educational events [3,5-8]. Notably, needs assessments at our institution identified lack of food as a barrier to attendance at MGR [2]. Complimentary food has also been associated with successful, well-attended internal medicine journal clubs [9].

Nevertheless, providing complimentary food requires substantial financial resources. However, before financial resources are allocated or sought from outside sources to provide free food, a study examining the effects of complimentary food on attendance at MGR should be undertaken. Attendee attitudes should also be assessed to evaluate whether this intervention changes one's attitude toward MGR [8]. To date, no systematic study has examined whether complimentary food increases attendance at MGR or what effect complimentary food has on attendee attitudes toward MGR.

MGR at Mayo Clinic (Rochester, Minnesota) is a 1-hour, noontime, weekly conference sponsored by the Department of Internal Medicine (DOM). The main site for MGR is a large auditorium in the outpatient clinic facility, but the conference is telecast elsewhere on campus. At Mayo Clinic, the principal objective of MGR is to educate DOM faculty, fellows, residents, and students about advances in internal medicine practice, research, and education. Additional objectives of MGR are to provide opportunities for socialization and CME credit. These objectives are similar to those of MGR in departments of medicine elsewhere [1-3,10].

We report the results of a prospective, before-and-after study of the effects of providing complimentary food on attendance at MGR. We also report the results of a survey that assessed the attitudes of MGR attendees on the provision of complimentary food.

## Methods

From September 25, 2002, to June 2, 2004, the attendance at MGR at Mayo Clinic (Rochester, Minnesota) was tracked by the use of head counts. During this period, food was available for purchase near the main auditorium and main telecast site. Beginning June 4, 2003, complimentary food was provided to MGR attendees at the main auditorium and at the main telecast site. The cost of the complimentary food was underwritten, in part, by an unrestricted grant from industry. Complimentary food included a sandwich, a piece of fruit, and a beverage. Attendance at MGR before complimentary food was provided was compared with attendance after complimentary food was provided. For data analysis, the periods were matched by week of the year because of possible seasonal variations that might affect attendance. Specifically, attendance at MGR during the pre-complimentary food period from September 25, 2002, (week 39 of 2002) to May 28, 2003, (week 22 of 2003) was compared with attendance at MGR during the complimentary food period from September 24, 2003, (week 39 of 2003) to June 2, 2004, (week 22 of 2004). Attendance counts were eliminated if attendance data were missing (eg, because of a holiday). Head count data were available for 29 corresponding weeks during the pre-complimentary food and complimentary food periods, for a total of 58 events in the analysis. Given the nonnormal distribution of the data, a Wilcoxon/Kruskal-Wallis test (rank sum test) was used to calculate *P *values. The level of significance was *P *< .05.

To assess the attitudes of MGR attendees about complimentary food, a Web-based survey was administered to 943 DOM faculty, fellows, and residents (See Appendix for the survey questions).

Group comparisons of survey response distributions were done using the Pearson χ^2 ^test. In cases of small cell counts, a hybrid Fisher exact test was used for the comparisons [11]. *P *values less than .05 were considered to be statistically significant. All analyses except the hybrid χ^2 ^analysis were conducted using JMP 5.1 software (SAS Institute, Inc, Cary, North Carolina). The hybrid χ^2 ^tests were run using a Fortran routine (The Fortran Company, Tucson, Arizona) on a UNIX platform (The Open Group, San Francisco, California) [12].

This study was approved by the Mayo Clinic Institutional Review Board.

## Results

### Effect of providing complimentary food on attendance at MGR

The mean (± SD) overall attendance increased 38.4% from 184.1 ± 90.4 per MGR session during the pre-complimentary food period to 254.8 ± 60.5 per MGR session during the complimentary food period (*P *< .001). Similar significant results were obtained when head count data for the main auditorium (*P *< .001) and the main telecast site (*P *< .001) were examined separately (Table 1). Notably, the size of the DOM increased by only 6% during the study period.

**Table 1:** Head Counts of Attendees at Medical Grand Rounds

	Head counts, average no. of attendees per MGR session*		
		
Site	Pre-complimentary food period	Complimentary food period	*P *value
Main auditorium	160.0 ± 81.6	208.6 ± 55.9	< .001
Telecast site	24.0 ± 12.5	46.2 ± 11.6	< .001
Total (both sites)	184.1 ± 90.4	254.8 ± 60.5	< .001

MGR, medical grand rounds.

* Mean ± SD.

### Complimentary food survey

After the study period, a Web-based survey was administered to all 943 DOM faculty, fellows, and residents. Of these, 444 (47.1%) responded (some respondents did not answer all questions; Tables 2, 3, 4, 5).

**Table 2:** Responses to the Question "On average, how frequently do you attend Medical Grand Rounds?"

	Respondents, %			
	
Response	Total* (*n *= 442)	Faculty†‡ (*n *= 261)	Fellow† (*n *= 74)	Resident‡ (*n *= 86)
Weekly	21.9	16.1	27.0	40.7
Monthly	45.5	42.5	50.0	50.0
4 to 6 times a year	28.1	35.6	18.9	9.3
1 to 2 times a year	3.8	5.0	2.7	0.0
Never	0.7	0.8	1.4	0.0

*Total includes respondents who were not physicians.

†Faculty vs fellows (*P *= .034).

‡Faculty vs residents (*P *< .001).

**Table 3:** Responses to the Question "Are you more or less likely to attend Medical Grand Rounds because of free food?"

	Respondents, %			
	
Response	Total* (*n *= 442)	Faculty†‡ (*n *= 261)	Fellow† (*n *= 74)	Resident‡ (*n *= 86)
Much more likely	30.3	21.8	51.4	44.2
Slightly more likely	39.8	44.1	28.4	38.4
Not at all	24.7	28.7	16.2	12.8
Less likely	2.3	1.9	2.7	3.5
Very unlikely	2.9	3.4	1.4	1.2

*Total includes respondents who were not physicians.

†Faculty vs fellows (*P *< .001).

‡Faculty vs residents (*P *< .001).

**Table 4:** Responses to the Question "How has your attendance at Medical Grand Rounds changed as a result of free food?"

	Respondents, %			
	
Response	Total* (*n *= 442)	Faculty†‡ (*n *= 262)	Fellow† (*n *= 73)	Resident‡ (*n *= 86)
Much more frequent	14.9	10.7	26.0	22.1
Slightly more frequent	38.7	35.9	42.5	47.7
Not at all	44.1	51.1	28.8	27.9
Less frequent	1.4	1.1	1.4	2.3
Much less frequent	0.9	1.1	1.4	0.0

*Total includes respondents who were not physicians.

†Faculty vs fellows (*P *= .003).

‡Faculty vs residents (*P *= .001).

**Table 5:** Responses to the Question "How would your attendance at Medical Grand Rounds change if food ceased being provided free of charge?"

	Respondents, %			
	
Response	Total* (*n *= 441)	Faculty†‡ (*n *= 262)	Fellow† (*n *= 74)	Resident‡ (*n *= 86)
Increase significantly	0.5	0.8	0.0	0.0
Increase somewhat	2.5	3.5	1.4	1.2
No change	44.0	51.5	28.4	25.6
Decrease somewhat	40.6	37.3	48.6	48.8
Decrease significantly	12.5	6.9	21.6	24.4

*Total includes respondents who were not physicians.

†Faculty vs fellows (*P *< .001).

‡Faculty vs residents (*P *< .001).

In response to the question "On average, how frequently do you attend MGR?" a majority of respondents (67.4%) reported that they attended either weekly or monthly. The responses of faculty members to this question were significantly different from the responses of fellows (*P *= .034) and residents (*P *< .001). Specifically, fellows and residents indicated that they attended MGR more frequently than did the faculty (Table 2).

In response to the question "Are you more or less likely to attend MGR because of free food?" 70.1% of the respondents indicated that they were more likely to attend, whereas only 5.2% indicated that they were less likely to attend. The faculty's responses to this question differed significantly from the fellows' responses (*P *< .001) and the residents' responses (*P *< .001). For example, only 21.8% of the faculty respondents indicated that they were "much more likely" to attend MGR because of complimentary food, compared with 51.4% of the fellows and 44.2% of the residents (Table 3).

In response to the question "How has your attendance at MGR changed as a result of free food?" 53.6% of the respondents indicated that their attendance had increased, whereas 44.1% indicated that their attendance had not changed at all. The faculty's responses to this question differed significantly from the fellows' responses (*P *= .003) and the residents' responses (*P *= .001). Compared with faculty respondents, more fellows and residents indicated that their attendance had increased "much more" and "slightly more" as a result of the complimentary food (Table 4).

In response to the question "How would your attendance at MGR change if food ceased being provided free of charge?" 53.1% of respondents indicated that their attendance would decrease, whereas 44.0% indicated that their attendance would not change. The faculty's responses to this question differed significantly from the fellows' responses (*P *< .001) and the residents' responses (*P *< .001). Compared with fellow and resident respondents, a much larger percentage of faculty respondents indicated that their attendance would not change. Compared with faculty respondents, more fellow and resident respondents indicated that their attendance would decrease (Table 5).

When asked if food is a distraction (eg, because of noise) at MGR, most respondents (80.3%) indicated that the food was not a distraction. This response was similar among residents, fellows, and faculty. Among all respondents, 81.7% disagreed with the following statement: "The DOM receives unrestricted support from industry to pay for food at MGR. As a result, industry representatives have influence over MGR."

## Discussion

Ours is the first systematic study assessing the effects of complimentary food on attendance at MGR. We found that, compared with attendance during the pre-complimentary food period, MGR attendance during the complimentary food period was significantly greater. These results suggest that providing free food may enhance attendance at MGR. The survey administered at the conclusion of the study period adds to these findings. A majority of respondents indicated that they were more likely to attend MGR because of the complimentary food and that their attendance increased because of it (although, compared with residents and fellow respondents, fewer faculty respondents reported that their attendance at MGR increased as a result of the complimentary food).

A number of factors (eg, program content and barriers to attendance) affect physician attendance at CME activities [13]. Survey data indicate that several barriers affect physicians' decisions to attend MGR, such as conflicting meetings, little presenter-attendee interaction, and inconvenient location [3]. Likewise, survey data indicate that some institutions provide complimentary food in an attempt to improve attendance at MGR [3]. Given that MGR occurs at noon at our institution, complimentary food not only removes a barrier (ie, by eliminating the need to choose between seeking food and attending MGR) but also adds an incentive for attending MGR (complimentary food). Some have described incentives as "the cornerstone of modern life" [14], and commonly acknowledged incentives for attending MGR, such as gaining new knowledge and CME credit, may not be sufficient for maintaining attendance. Although some view incentives negatively, incentives can be effective [5,6,8,14]. Therefore, as one examines strategies to increase attendance at MGR, one should consider not only removal of barriers but also the effect of incentives.

Although providing complimentary food may be associated with increased attendance at MGR, it also increases the cost of conducting MGR, which, for many departments, is the most expensive conference to conduct [3]. The cost of providing complimentary food at MGR at our institution is approximately $60,000 per year. To defray these costs, many departments, including ours, have garnered industry (eg, pharmaceutical) financial support [1-3,15,16].

Industry support of MGR raises the ethical concern of industry influence over MGR organizers, content, speakers, and attendees [1-3,17,18]. This concern can be addressed by using the following guidelines: 1) industry support should be unrestricted; 2) MGR speakers should disclose to attendees any conflicts of interest; 3) industry representatives should not determine MGR content; and 4) presentations at MGR should be unbiased, especially when the industry sponsor's products are discussed [3,19,20]. These guidelines are rigorously followed at our institution. Notably, our MGR attendees did not perceive inappropriate industry influence over the conference. However, measuring the influence of industry support by self-report may be biased. A recent study concluded that physicians' attitudes regarding industry support of CME activities may be biased (ie, those attending industry-supported activities are less likely to report bias than those attending non-industry-supported activities) [21].

Our study has several limitations. Although we used a prospective, before-and-after design, our study was neither randomized nor blinded. However, such a design would have been impractical. We could not control for or compare the quality of presentations during the 2 study periods, and, therefore, we do not know whether this factor contributed to an increased attendance at the MGR sessions. However, we compared attendance data matched for time of year to minimize bias (eg, related to holidays). Furthermore, advertisement of MGR did not change during the 2 study periods. We did not change the time of day or the day of the week that MGR was held during the study period. In addition, although our survey data suggest that faculty, fellows, and residents may behave differently in response to complimentary food as an incentive for attending MGR, we were unable to break down the attendance data according to attendee training status. Furthermore, we were unable to break down the attendance data by physician versus nonphysician attendees. Finally, although providing complimentary food at MGR at our institution was associated with increased attendance, our results may not be generalizable to other institutions. Likewise, care should be taken when applying our results to other educational activities. Future research should address these limitations.

## Conclusion

Increased attendance at MGR occurred after complimentary food was provided to MGR attendees. Our data suggest that faculty, fellows, and residents are more likely to attend MGR if complimentary food is provided and less likely to attend if complimentary food is not provided. Most attendees do not perceive the complimentary food to be a distraction, nor do they perceive inappropriate industry influence over the conference. Providing complimentary food may be an effective strategy for increasing attendance at MGR.

## Abbreviations

CME, continuing medical education

DOM, Department of Medicine

MGR, medical grand rounds

## Competing interests

The author(s) declare that they have no competing interests.

## Authors' contributions

PSM, NFL, SCL, AT, and TMH conceived of the study. CMS, PSM, and MLR carried out the literature review. CMS, PSM, NFL, SCL, AT, and TMH designed the survey evaluating attendee attitudes. CMS compiled the data and performed the statistical analyses. CMS, PSM, MLR, NFL, SCL, AT, and TMH prepared and reviewed the manuscript. All authors read and approved the final manuscript.

## Appendix 1 – Survey Questions

In addition to indicating their position (ie, faculty, fellow, or resident), respondents were asked the following questions about medical grand rounds (MGR) (with accompanying choices):

1) On average, how frequently do you attend MGR? (weekly, monthly, 4 to 6 times per year, 1 to 2 times per year, never)

2) Are you more or less likely to attend MGR because of free food? (much more likely, slightly more likely, not at all, less likely, very unlikely)

3) How has your attendance at MGR changed as a result of free food? (much more frequent, slightly more frequent, not at all, less frequent, much less frequent)

4) How would your attendance at MGR change if food ceased being provided free of charge? (increase significantly, increase somewhat, no change, decrease somewhat, decrease significantly)

5) Indicate your agreement with the following statement: Food is a distraction (eg, due to noise) at MGR. (strongly agree, agree, neither agree nor disagree, disagree, strongly disagree)

6) Indicate your agreement with the following statement: The Department of Medicine receives unrestricted support from industry to pay for food at MGR. As a result, industry representatives have influence over MGR (eg, speaker choice and content). (strongly agree, agree, neither agree nor disagree, disagree, strongly disagree)

## Pre-publication history

The pre-publication history for this paper can be accessed here:



## References

[B1] Hebert RS, Wright SM (2003). Re-examining the value of medical grand rounds. Acad Med.

[B2] Mueller PS, Litin SC, Sowden ML, Habermann TM, LaRusso NF (2003). Strategies for improving attendance at medical grand rounds at an academic medical center. Mayo Clin Proc.

[B3] Mueller PS, Segovis CM, Litin SC, Habermann TM, Parrino TA (2006). Current status of medical grand rounds in departments of medicine at US medical schools. Mayo Clin Proc.

[B4] Moore DE, Davis D, Barnes BE, Fox R (2003). A framework for outcomes evaluation in the continuing professional development of physicians. The continuing professional development of physicians: from research to practice.

[B5] Dhatariya K (2003). Improving attendance at medical grand rounds [letter]. Mayo Clin Proc.

[B6] Mueller PS, Litin SC, Sowden ML, Habermann TM, LaRusso NF (2003). Improving attendance at medical grand rounds [letter]. Mayo Clin Proc.

[B7] Reinharth D (2003). Improving attendance at medical grand rounds [letter]. Mayo Clin Proc.

[B8] Buckman R Gravy train: investors in Hong Kong eat up annual meetings; elderly shareholders will go where the food is best;rushing the buffet table. Wall Street Journal.

[B9] Sidorov J (1995). How are internal medicine residency journal clubs organized, and what makes them successful?. Arch Intern Med.

[B10] Parrino TA, White AT (1990). Grand rounds revisited: results of a survey of U.S. Departments of Medicine. Am J Med.

[B11] Mehta CR, Patel NR (1986). A hybrid algorithm for Fisher's exact test in unordered r*c contingency tables. Communications in Statistics A, Theory and Methods.

[B12] Mehta CR, Patel NR (1986). Algorithm 643. FEXACT: a Fortran subroutine for Fisher's exact test on unordered r*c contingency tables. ACM Transactions on Mathematical Software.

[B13] Moore D, Bennett N, Knox A, Kristofco R, Davis DA, Fox RD (1994). Participation in formal CME: factors affecting decision-making. The physician as learner: linking research to practice.

[B14] Levitt SD, Dubner SJ (2005). Freakonomics: a rogue economist explores the hidden side of everything.

[B15] (2002). Medical education service suppliers 2002 review. Med Mark Med.

[B16] Holmer AF (2001). Industry strongly supports continuing medical education. JAMA.

[B17] Relman AS, ACCME (2003). Defending professional independence: ACCME's proposed new guidelines for commercial support of CME. JAMA.

[B18] Alpert JS (2005). Doctors and the drug industry: how can we handle potential conflicts of interest?. Am J Med.

[B19] American Medical Association, Council on Ethical and Judicial Affairs (2002). Code of medical ethics: current opinions with annotations 2002–2003.

[B20] Coyle SL, Ethics and Human Rights Committee, American College of Physicians-American Society of Internal Medicine (2002). Physician industry relations. Part 2: organizational issues. Ann Intern Med.

[B21] Mueller PS, Hook CC, Litin SC (2007). Physician preferences and attitudes regarding industry support of CME programs. Am J Med.

